# Molecular Mechanisms Underlying Inhibitory Binding of Alkylimidazolium Ionic Liquids to Laccase

**DOI:** 10.3390/molecules22081353

**Published:** 2017-08-15

**Authors:** Jianliang Sun, Hao Liu, Wenping Yang, Shicheng Chen, Shiyu Fu

**Affiliations:** 1 State Key Laboratory of Pulp and Paper Engineering, South China University of Technology, Guangzhou 510640, Guangdong, China; sunjianliang0102@163.com (J.S.); shyfu@scut.edu.cn (S.F.); 2 School of Mathematics, South China University of Technology, Guangzhou 510640, Guangdong, China; scwpyang@scut.edu.cn; 3 Department of Microbiology and Molecular Genetics, Michigan State University, East Lansing, MI 48824, USA; shicheng@msu.edu

**Keywords:** ionic liquids (ILs), laccase, alkylimidazolium cations, competitive binding, kinetics, Hofmeister effects, molecular simulation

## Abstract

Water-miscible alkylimidazolium ionic liquids (ILs) are “green” co-solvents for laccase catalysis, but generally inhibit enzyme activity. Here, we present novel insights into inhibition mechanisms by a combination of enzyme kinetics analysis and molecular simulation. Alkylimidazolium cations competitively bound to the T_I_ Cu active pocket in the laccase through hydrophobic interactions. Cations with shorter alkyl chains (C_2_~C_6_) entered the channel inside the pocket, exhibiting a high compatibility with laccase (competitive inhibition constant K_ic_ = 3.36~3.83 mM). Under the same conditions, [Omim]Cl (K_ic_ = 2.15 mM) and [Dmim]Cl (K_ic_ = 0.18 mM) with longer alkyl chains bound with Leu296 or Leu297 near the pocket edge and Leu429 around T_I_ Cu, which resulted in stronger inhibition. Complexation with alkylimidazolium cations shifted the pH optima of laccase to the right by 0.5 unit, and might, thereby, lead to invalidation of the Hofmeister series of anions. EtSO_4_^−^ showed higher biocompatibility than did Ac^−^ or Cl^−^, probably due to its binding near the T_I_ Cu and its hindering the entry of alkylimidazolium cations. In addition, all tested ILs accelerated the scavenging of 2, 2′-azino-bis-(3-ethylbenzothiazoline-6-sulphonic acid) (ABTS) radicals, which, however, did not play a determining role in the inhibition of laccase.

## 1. Introduction

**Figure 1:**
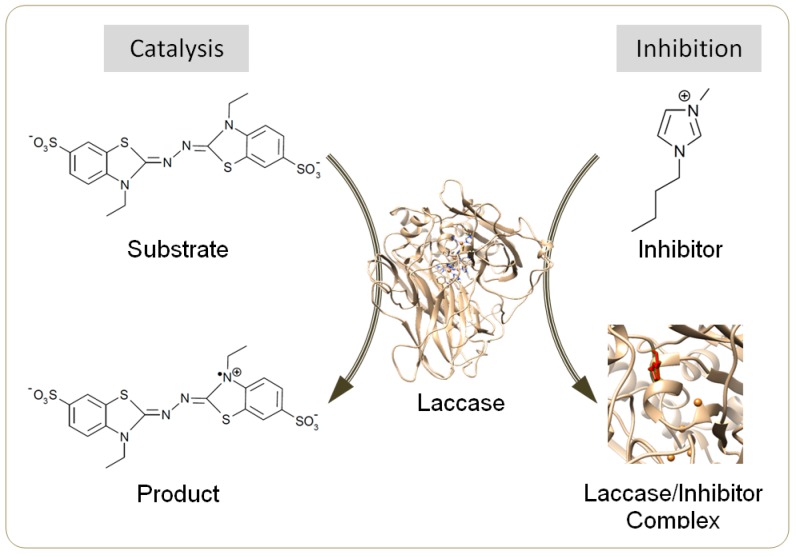
Scheme for catalysis by laccase and for inhibition by ionic liquids (ILs).

Laccase (EC 1.10.3.2) is an efficient biocatalyst for synthesizing dimers, polymers and composites from a diverse range of substrates including phenolics, aromatic amines, flavonoids, acrylamide and thiols [1,2]. Laccase-induced synthesis is often conducted in aqueous mixtures of organic solvents, e.g., acetone and methanol, to promote the yield and/or molecular weight of products [3,4]. Many laccases are tolerant to these solvents at a high content, e.g., 50% (*v*/*v*) [4,5]. However, the use of conventional volatile solvents threatens the environment [6]. Recently, ionic liquids (ILs) have been revealed as ideal alternative co-solvents due to their negligible vapor pressure, remarkable thermal stability and excellent recyclability [6,7]. Selected ILs could further lessen the inactivation of laccase by the template molecules during aniline polymerization [8], or by the oxidative form of redox mediators [9]. However, most water-miscible ILs for potential use in homogeneous synthesis have been identified as laccase inhibitors (Figure 1) except several particular species, e.g., [Emim]EtSO_4_ and [TMA]TfO, under specified conditions [8,10,11,12].

Similar to those typical inorganic salts, ILs are constituted of a pair of cation and anion moieties. Their biocompatibilities are greatly dependent on physicochemical properties [7]. For instance, *Trametes versicolor* (*Tve*) laccase was more frequently supported by hydrophobic ILs [10]. The inactivation effects of laccase by alkylimidazolium ILs aggravated with the increase of alkyl chain length [13,14]. On the contrary, there is evidence showing that alkyl sulfate anions with shorter chain length have less inhibitory effects on *Tve* laccase activity [10]. For a better explanation of the distinct effects of homologous series of ILs on enzyme stability, the Hofmeister effects hypothesis has proposed; in brief, the loss of enzyme activity tends to occur in the presence of chaotropic anions, e.g., [NTf_2_]^−^>[SCN]^−^>[CF_3_SO_3_]^−^>Br^−^>[BF_4_]^−^>Cl^−^>Ac^−^>SO_4_^2−^>PO_4_^3−^, and kosmotropic cations, e.g., Na^+^>K^+^> [Hmim]^+^>[BmPy]^+^>[Bmim]^+^>[Emim]^+^>[N1,1,1,1]^+^ [15,16]. Anions exhibit stronger Hofmeister effects than cations [11]. The above Hofmeister series is an empirical rule developed on the basis of biochemical assays. It is not universally valid for predicting effects of ILs’ structures on any enzyme due to the wide variations of enzyme sources, substrate species and media features [15,16]. To develop a better understanding, it is important to directly explore the laccase/salt molecular interactions and characterize the respective role of cations and anions. Previous authors have only reported the change in kinetics parameters of laccase by addition of ILs [5,10,11,12,13,14]. Advanced investigation tools such as in silico simulation have been used for analyzing the molecular interactions between laccase and substrate, in particular at the binding sites [12,17]. These tools, however, have not been applied in the study of laccase/IL binding. This work investigated the inhibition mechanisms of water-soluble alkylimidazolium ILs on *Myceliophthora thermophila* (*Mth*) laccase by using molecular docking and kinetics methods. The validation of the Hofmeister series, the pH optima shift of laccase and radical scavenging of oxidation products in the presence of ILS were all comprehensively discussed.

## 2. Results

### 2.1. Competitive Binding of Alkylimidazolium Cations

**Figure 2:**
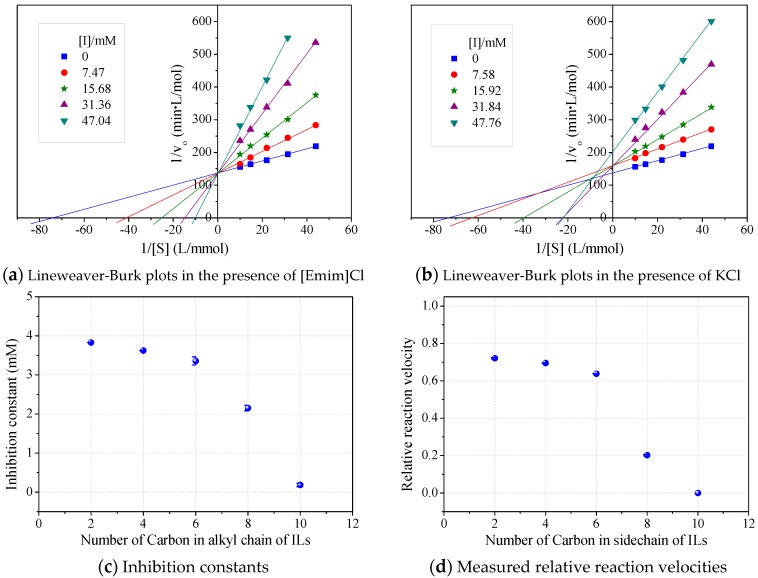
Effects of alkyl chain length in alkylimidazolium cations on kinetics of laccase/ABTS oxidation at pH 3. (Note: Standard deviation bars were from triplicate measurements).

The roles of cations were revealed by comparing the kinetics parameters, i.e., initial velocity (v_o_) and inhibition constants (K_i_), and the docking sites of five alkylimidazolium chlorides ([C_n_mim]Cl, *n* = 2, 4, 6, 8, 10, Table 1). All the tested ILs lowered laccase activity through competitive binding as indicated by the Lineweaver–Burk plots of 1/v_o_ vs. 1/[S] ([S] was the concentration of substrate) (Figure 2a and Figure S1). All plots obtained at varied concentrations of IL ([I]) intersected at the same point on the vertical axis. The apparent Michaelis constant (K_m_) increased with increasing [I] while the maximum velocity (V_max_) remained constant. Competitive inhibition constants (K_ic_) were thereby calculated from Equation (2) and shown in Figure 2c. Clearly, K_ic_ value decreased with the increase of alkyl chain length or n value. In particular, a dramatic inhibition was detected when *n* > 6. For [Omim]Cl (*n* = 8) or [Dmim]Cl (*n* = 10), the K_ic_ was determined as 2.15 and 0.18 mM, respectively. Lower K_ic_ corresponded to stronger inhibition capacity. The reaction velocities under regular conditions (See Section 4.4) were measured to verify the variation trend of K_ic_. The results in Figure 2d showed that laccase was almost completely inactivated by 50 mM of [Dmim]Cl. However, [Emim]Cl, [Bmim]Cl and [Hmim]Cl with shorter alkyl chains retained 65~75% of laccase activity, indicating that they are more promising for practical use compared to [Dmim]Cl or [Omim]Cl.

**Figure 3:**
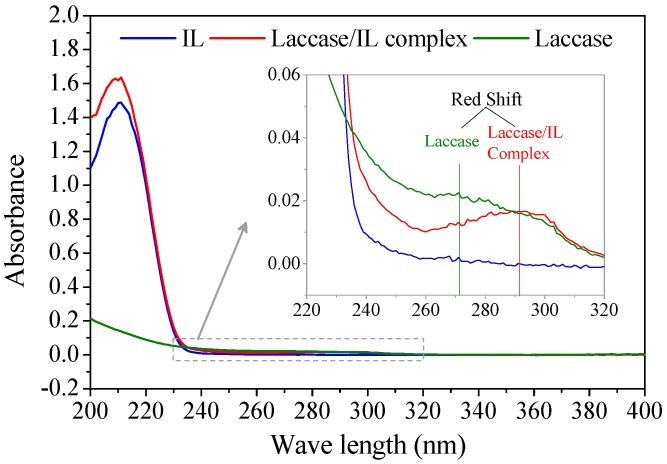
UV/Vis spectra of [Bmim]Cl (IL), laccase and laccase/[Bmim]Cl complex.

According to enzyme kinetics theory, competitive binding means that the inhibitors irreversibly occupy the specific binding sites for substrates and form an enzyme–inhibitor complex [18]. The formation of a laccase/IL complex was verified with UV/Vis spectroscopy (Figure 3). A laccase/IL complex sample was obtained by soaking *Mth* laccase in [Bmim]Cl/buffer solution, followed with lyophilization and thorough washing with absolute ethanol. The solid resultant was totally re-dissolved in water. The UV/Vis spectrum of the complex had both specific absorption bands of [Bmim]Cl (at around 210 nm) and *Mth* laccase (260~300 nm). There was obvious red shift from the absorption peak of laccase protein at 272 nm to the peak of laccase/[Bmim]Cl complex at 292 nm, which suggests the complexation of laccase with [Bmim]Cl absorbed energy. In addition, the re-dissolved complex still retained 65% of the initial activity (data not shown).

**Figure 4:**
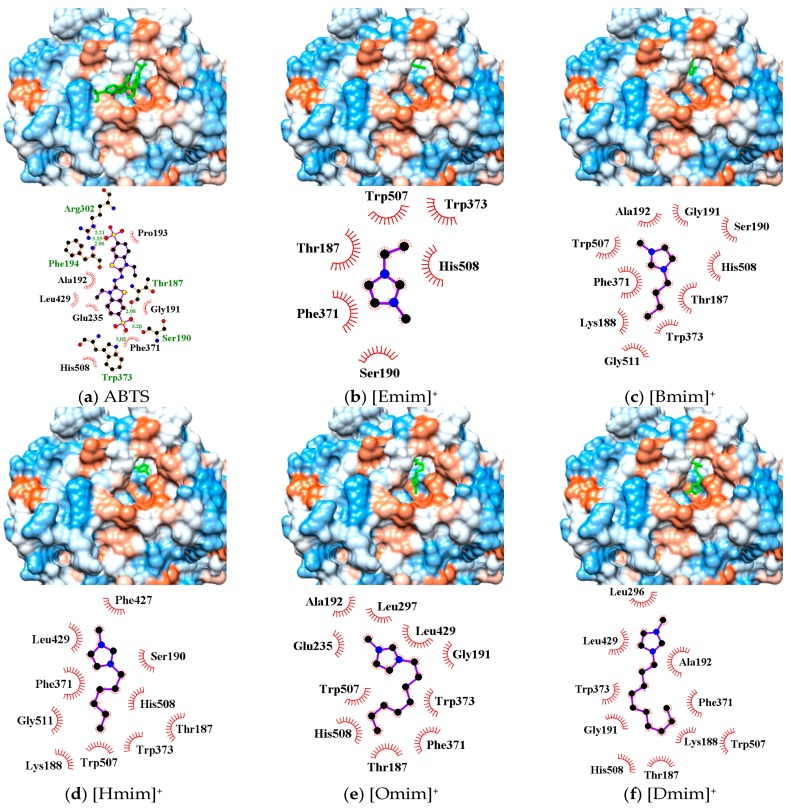
Three dimensional conformations and binding sites from the docking of ABTS (**a**) and five alkylimidazolium cations (**b**–**f**) to the T_I_ Cu active pocket of *Mth* laccase.

In silico simulation showed that the active pocket near the T_I_ Cu site (His431, Cys503, His508) in *Mth* laccase could accommodate substrate molecules such as 2, 2′-azino-bis-(3-ethylbenzothiazoline-6-sulphonic acid) (ABTS) and three other phenolics (Figure 4a and Figure S2). The interactions between ABTS and binding sites were maintained by (1) hydrogen bonds at Thr187, Ser190, Phe194, Arg302 and Trp373, and (2) hydrophobic force at Gly191, Ala192, Pro193, Glu235, Phe371, Leu429 and His508.

The active pocket around the T_I_ Cu also functioned as the optimal binding site for alkylimidazolium cations (Figure 4b–f). All cations could enter the pocket in the same orientation. The methylimidazolium head possessing positive charge pointed at the hydrophobic outer edge of the pocket (brown color), while the alkyl chain stretched into the hydrophilic inner channel (blue color). Alkylimidazolium cations complexed with laccase dominantly through hydrophobic interactions. The binding sites, Thr187, Ser190/Gly191, Phe371, Trp373 and His508, were identical for all the tested inhibitors as well as the substrate such as ABTS. Due to the lack of proton acceptor or donor groups in alkylimidazolium structure, there was no hydrogen bond formed between laccase and IL cations. Electrostatic interactions might exist and affect the orientation. Figure 4 showed that the positive head was always apart from His508, a base residue, probably due to the electrostatic repulsion. On the other hand, small cations such as [Emim]^+^ could entirely enter the inner channel in the pocket, probably leaving sufficient space for ABTS binding near the T_I_ Cu site. [Omim]^+^ and [Dmim]^+^ occupied Leu296/Leu297 near the pocket edge and Leu429 around T_I_ Cu, causing more server stereo-hindrance. As a result, the laccase activity was strongly or almost completely inhibited (Figure 2). Although Hofmeister effects hypothesis involves the cation series, our findings revealed the importance of stereo-hindrance effect especially the occupying of key amino acids in the IL-induced inhibition.

**Figure 5:**
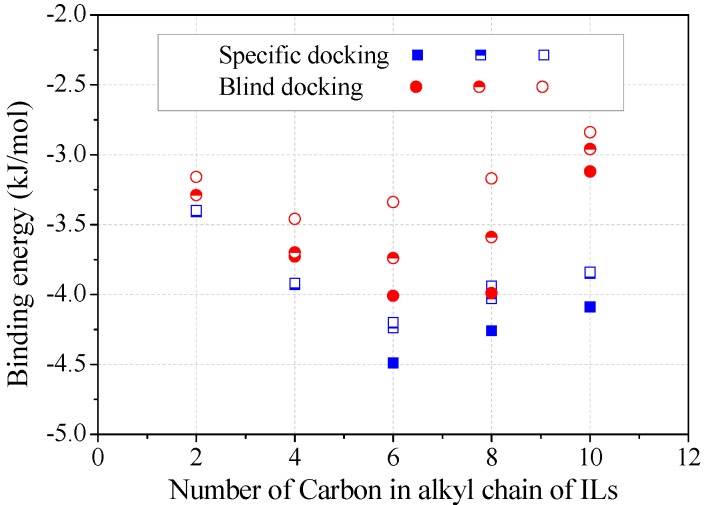
Binding energy varied with the alkyl chain length of alkylimidazolium cations. (Note: The top three docking conformations were represented as filled, half-filled and empty symbols, respectively).

The binding energy was estimated from the top three trial conformations (Figure 5). Obviously, specific docking gave more reliable results than blind docking for achieving energy minimization, although these two operations resulted in the same binding sites for alkylimidazolium cations. The decrease of negative binding energy from C_2_ to C_6_ corresponded to the increase in the stability of enzyme–inhibitor complex, consistent with the inhibition kinetics (Figure 2c). Binding energy slightly increased from C_6_ to C_8_, according to which less inhibition was expected. Many authors have also correlated the binding energy predictions with the inhibition capacities of inhibitors [17,19]. However, due to the occupying of several key amino acids, the inhibition by [Omim]^+^ or [Dmim]^+^ was rather significant.

### 2.2. Hofmeister Series of IL Anions

**Figure 6:**
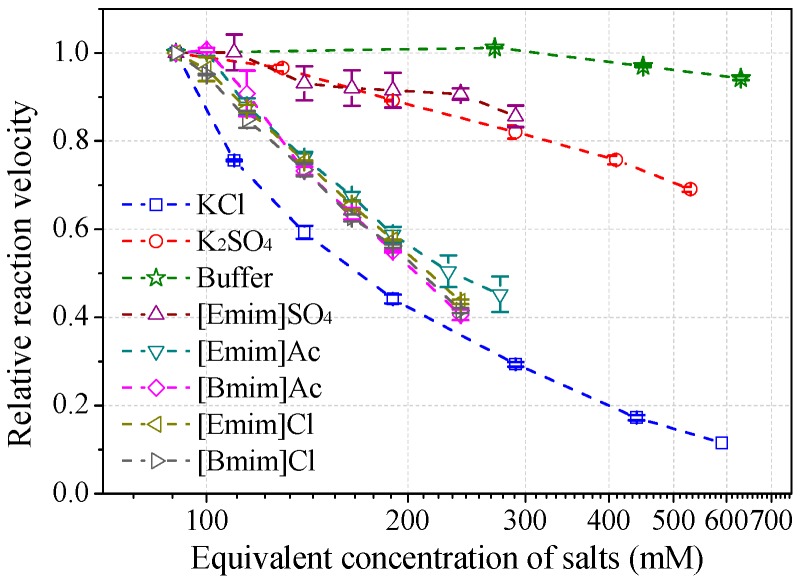
Salt effects on laccase activity towards ABTS at pH 3. (Note: 30 mM buffer was used to maintain the pH for all salts, normalized with the buffer control).

Effects of salt species at various concentrations on laccase activity were studied to characterize the role of IL anions. The results (Figure 6) demonstrated that anions play a central role in the salt-induced laccase inactivation, regardless of whether the cation is organic or inorganic. Mixed triacid bases, i.e., phosphate and citrate, were the most compatible species, retaining at least 90% of activity at a concentration of 200 mM (or equivalent molar concentration of 600 mM). [Emim]EtSO_4_ and K_2_SO_4_ ranked second, showing similar high kosmotropic (hydrated) features. Instead, acetate and chloride ILs performed more chaotropic (weakly hydrated) as stronger inhibitors. For example, near 50% of laccase activity was lost by 100~130 mM acetate or chloride ILs at pH 3. KCl caused more activity loss than alkylimidazolium chlorides.

**Figure 7:**
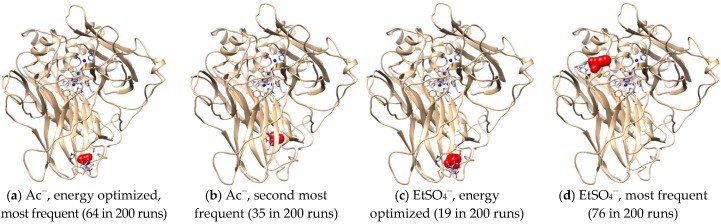
Optimal docking conformations for Ac^−^ and EtSO_4_^−^. (Note: Blue spheres are Cu ions; red structures are IL anions).

Intermolecular interactions between laccase and IL anions were built up for a better understanding of the Hofmeister series. Figure 7a,c showed that Ac^−^ and EtSO_4_^−^ favorably bound with the same sites in *Mth* laccase, forming hydrogen bonds with Thr246 and Arg281 and hydrophobic interactions with His245. The optimal binding energy for Ac^−^ and EtSO_4_^−^ were −3.42 and −4.06 kcal/mol, respectively. This site was also the most frequent one for Ac^−^ among 200 docking runs (Figure 7a). However, together with several other predicted sites (Figure 7b), it was far away from the specific sites composed of four Cu ions. On the contrary, the most frequent binding sites for EtSO_4_^−^ were very close to the T_I_ Cu active pocket (Figure 7d). This anion could thereby alter the ionic charge around the active site, change the surrounding static electric field and finally prevent the entry of alkylimidazolium cations into the pocket.

### 2.3. Shift of Laccase Optimal pH by ILs

**Figure 8:**
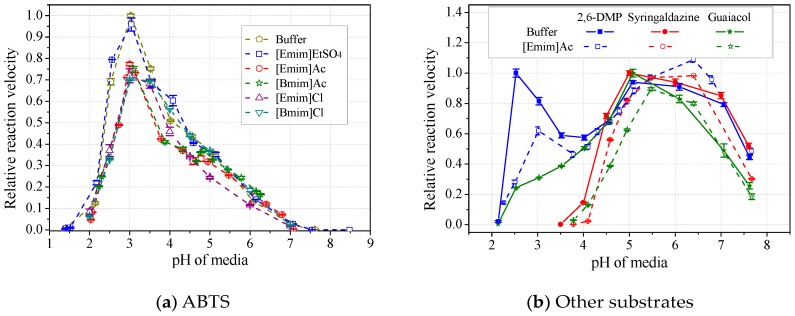
Effects of ILs on pH profiles of laccase activity towards ABTS (**a**) and other substrates (**b**). (Note: Normalized with respect to the maximum in buffer).

The formation of the laccase/IL complex would possibly alter the enzyme’s pH optima. Results in Figure 8a showed that the laccase pH optima towards ABTS was not affected by any tested IL, after calibration of media pH. Loss of laccase activity was up to 30%, following an order of [Emim]EtSO_4_<[Emim]Ac<[Bmim]Ac<[Emim]Cl<[Bmim]Cl. However, when using 2,6-DMP, syringaldazine or guaiacol as substrate, laccase’s optimal pH was usually slightly shifted to the right by 0.5 unit in the presence of alkylimidazolium ILs (Figure 8b and Table 2). The shift of pH optima resulted in either depression or activation of laccase, depending on both the species of ILs and substrates. In particular, for laccase activity towards 2,6-DMP, the shift of the second pH optima (5~6.5) by ILs led to apparent activation instead of inhibition, contradicting the Hofmeister series. Therefore, the substrate species and possible pH optima shifts should be considered in the study of the Hofmeister series. Table 2 also showed that acetate ILs induced more shifts than those paired with strong anions. [Emim]EtSO_4_ had better ability to maintain the optimal pH than did other species, showing the highest compatibility with laccase.

### 2.4. Scavenging of ABTS Cation Radicals by ILs

**Figure 9:**
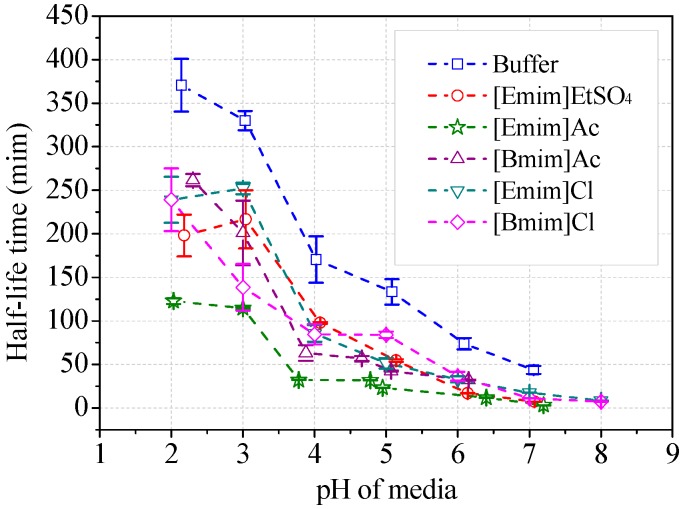
Stability of ABTS cation radicals in ILs/buffer media. (Note: Initial concentration of ABTS was 10 μM).

The stability of ABTS cation radicals (ABTS^+^) was tested with addition of alkylimidazolium ILs (Figure 9). The radicals were relatively stable in acidic buffer (30 mM), i.e., pH 2~3. The scavenging rate of ABTS^+^ increased with the increasing media pH. The half-life decreased from 370 min at pH 2 to 43.7 min at pH 7, which did not influence the measurement of laccase activity (usually taking 1~3 min). The addition of alkylimidazolium ILs (50 mM) in the buffer remarkably accelerated the elimination of ABTS^+^ (Figure 9). [Emim]Ac showed the strongest anti-radical activities. The half-life was decreased with 50 mM [Emim]Ac to 122 min at around pH 2 and to 4.3 min at pH 7. When measuring the reaction velocity in [Emim]Ac/buffer media within 2 min, the apparent laccase activity was 3.4% lower than the theoretical value due to the scavenging of ABTS^+^, given that [Emim]Ac has no inhibition to laccase. However, the measured inhibition rate by [Emim]Ac was 83.6% according to Figure 8a. The other four IL species exhibited weaker capacities to transform ABTS^+^. Collectively, they lowered the half-life to 7~8 min at neutral pH and caused, at most, 2.1% of apparent activity lost. At optimal pH for ABTS, the half-lives of ABTS^+^ were all higher than 85 min, suggesting that the loss of apparent activity was minimal, within the measurement error range. Therefore, the scavenging of ABTS^+^ did not play a dominant role in the inhibitory effects of ILs on laccase/ABTS reaction. Accordant results were observed for 2,6-DMP. The brown color of the oxidative radicals was faded in the presence of ILs as shown in Figure S3. The scavenging properties of other radicals from syringaldazine and guaiacol by ILs were not measured because these radicals have a high tendency to couple with each other, and are impossible to isolate.

## 3. Discussion

### 3.1. Inhibitory Binding

The inhibition of laccases by alkylimidazolium ILs has been previously investigated by measuring apparent kinetics parameters (v_o_, V_max_ and K_m_) or residual enzyme activities (mostly expressed as half-life). However, the mechanisms underlying the kinetics and molecular interactions remained unexplored. Our results indicated that a competitive inhibition mechanism existed in the interactions between *Mth* laccase and alkylimidazolium chlorides (Figure 2). Spectroscopic characterization verified the formation of the laccase/IL complex (Figure 3). Molecular simulation (Figure 4) further indicated that all the water-miscible alkylimidazolium ILs possibly formed competitive binding with *Mth* laccase. This study will direct the engineering of laccase, for example, for obtaining novel variants compatible with lignin-dissolving ILs for lignocellulosic biorefinery. On the other hand, the IL-induced inhibition features may vary with the enzyme species. For instance, the binding of [Bmim]BF_4_ to horseradish peroxidase, another species of polyphenol oxidase, has been designated as a non-competitive situation [20]. Simulation work deserves to be done to reveal the difference in molecular interactions between laccase and other polyphenol oxidases.

Different from alkylimidazolium chlorides, KCl triggered severer depression of *Mth* laccase activity than ILs (Figure 2b), probably through mixed mechanisms (competitive/non-competitive). K^+^ could bind to laccase flexibly at both specific and non-specific sites. Raseda et al. [21] investigated the effects of NaCl on *Tve* laccase activity towards ABTS (at pH 3) and also found dual inhibition mechanisms. The competitive constant, K_ic_, and the uncompetitive constant, K_iu_, were 0.35 and 18.1 mM, respectively. Therefore, competitive inhibition was the dominant reason of the laccase inhibition by chloride salts [21].

### 3.2. Hofmeister Effects

Results in this work revealed new insights into Hofmeister effects. The alkylimidazolium cations competitively bound to the T_I_ Cu active pocket of *Mth* laccase. Those species with long alkyl chains formed a stereo-hindrance to regular substrate molecules such as ABTS. Significant inhibition was expected when several key amino acids (i.e., Leu296/Leu297 and Leu429) were occupied. The free paired anions in ILs may loosely attach to enzyme surface areas. These bound anions could trap protons from media, which contributed to the right shift of laccase optimal pH. In particular, EtSO_4_^¯¯^ could bind near the T_I_ Cu active pocket, alter the distribution of ionic charge around the active pocket, and finally hinder the binding of alkylimidazolium cations into the pocket.

The theory of the Hofmeister series was successfully applied to predict the stability of proteins in the presence of a variety of salts. Similar Hofmeister series of alkylimidazolium cations were demonstrated by several different studies [13,22]. For instance, the hydrophobic force was the dominant interaction between alkylimidazolium cations and hemeprotein hemoglobin, which also gradually increased with the increase in the cation chain length [22]. In addition, the Hofmeister series of ILs anions may not be universally valid. For example, the stability and activity of lysozymes were greatly promoted by both SO_4_^−^ and Ac^−^ [23]; meanwhile, these two anions generally inhibit the activity of laccase. The above results suggest that different enzymes may have different Hofmeister effects of ILs [24]. Our results suggest that it is important to perform integrated analysis of enzyme kinetics and molecular simulations to build up quantitative and qualitative concepts of the Hofmeister series.

### 3.3. Optimal pH Shift

Shifts in enzymes’ optimal pH by salts were observed in several early studies, e.g., HSO_3_^−^ on ATPase [25], Mg^2+^ on ribulose bisphosphate carboxylase [26]. Recently, Yang et al. [27] reported that the optimal pH of tyrosinase was right shifted by 0.5 unit with 2% [Bmim]MeSO_4_ and 2% [Bmim]BF_4_, while not being affected by [Bmim]PF_6_. Another study showed that the optimal pH of alkaline phosphatase was left shifted by Na^+^ and K^+^ salts [28]. For laccase, there was only indirect evidence that *Tve* laccase could be activated by [Mmim]MeSO_4_ at elevated pHs, i.e., 7 and 9 [29]. Assumptions about pH optima shifts were proposed in our previous article [30] and confirmed in this work.

Shifts in optimal pH were probably due to the formation of the laccase–IL complex. Given that cations bind to enzyme active sites, the paired anions are hydrolyzed to generate OH^−^ in surrounding circumstance. Thus, the apparent optimal pH shifts to right. [Emim]EtSO_4_ exhibited a good compatibility with laccase, probably due to its difficulty in forming the laccase/[Emim]^+^ complex. Then, the pH optima of laccase were less affected by [Emim]EtSO_4_ (Table 2). Another possible mechanism is that complexation with IL ions may alter the isoelectric point of a protein due to the change in static electric field in protein molecules. However, a further investigation is warranted to better understand the pH optima affected by these salts.

Our results showed that the change in laccase optimal pH may be linked to ILs’ hydrolyzabilities. Acetate ILs induced more shifts than those paired with strong anions. [Emim]Ac dissociated insufficiently into isolated ions in aqueous solution [31]. It acted as a basic IL and could be acidified with pressurized CO_2_ [32]. Our results in Figure S4 showed that alkylimidazolium acetates probably functioned as a weak-acid/weak-base salt similar to NH_4_Ac, since pH was decreased in neutral buffer but promoted in acidic buffers. The additional OH^−^ and H^+^ were generated from the hydrolysis of acetate anions and alkylimidazolium cations (Formulas (1)–(3)), respectively, as shown in the following formula.

(1) $$\left[\mathrm{Emim}\right]\mathrm{Ac}\Leftrightarrow {\left[\mathrm{Emim}\right]}^{+}+{\mathrm{Ac}}^{-}$$

(2) $${\mathrm{Ac}}^{-}+H_{2}O\Leftrightarrow \mathrm{HAC}+{\mathrm{OH}}^{-}$$

(3) $${\left[\mathrm{Emim}\right]}^{+}+H_{2}O\Leftrightarrow \left[\mathrm{Emim}\right]\mathrm{OH}+H^{+}$$

### 3.4. Radicals Scavenging

Both cations and anions of ILs diminished the blue color of ABTS^+^ in experiments. The radical scavenging was thereby studied as an inhibition pathway. Our previous work showed that ABTS^+^ could be transformed into a reductive state by many common organic solvents [33]. For example, 50% reduction of ABTS^+^ occurred in 80% acetone aqueous solution within 7 min, while no change was detected in 80% acetic acid, suggesting the stabilization of ABTS^+^ by acid [33]. Liu et al. [12] found that the stability of the ABTS^+^ was higher in the presence of [Emim]EtSO_4_ than [Emim]MeSO_4_, [Emim]Ac, or [Emim]DMP at pH 4.5, which was in accordance with our results. Eshtaya et al. [34] also reported the compatibility of [Emim]EtSO_4_ with ABTS^+^ and used them in combination for electrochemical oxidation of lignin. In this work, the contribution of ABTS^+^ scavenging was found to not be a critical factor in LS-induced inhibition; however, it should be taken into consideration when using the laccase/ABTS system for longstanding reactions.

## 4. Materials and Methods

### 4.1. Chemicals and Reagents

All ionic liquids were purchased from Sigma-Aldrich (St. Louis, MO, USA). ABTS (≥99% purity, HPLC grade) was purchased from Fluka biochemika (Steinheim, Germany). 2,6-DMP (≥98%) was from Aladdin Chemistry Co., Ltd. (Shanghai, China). Guaiacol (≥99%, food grade) and syringaldazine (≥99%) were from Sigma-Aldrich. All other chemicals were of analytical grade and were obtained from Guangzhou Chemical Reagent Factory (Guangzhou, China). Double-distilled water was used to prepare solutions.

### 4.2. Laccase and Homologous Modeling

Laccase (Novozym 51003) was supplied by Novozymes (Tianjin, China) Biotechnology Co., Ltd. (Tianjin, China). This enzyme was produced in *Aspergillus oryzae* by heterologous expression of a laccase gene from *M. thermophila*. The amino acid sequence (Figure S5a) was obtained from a patent file (No. US 7622287B2) assigned to Novozymes A/S in 2009. The sequence was uploaded to the online Swiss-Model server (http://www.swissmodel.expasy.org) for modeling. The template 1gw0, a *Melanocarpus albomyces* (*Mal*) laccase, was the most favorable one with a sequence identity of 74.16%. The 3D model (Figure S5b) was then constructed by the online server, showing a GMQE score of 0.92, near the ideal value 1, and a QMEAN4 score of 0.27, close to the ideal value 0 (Figure S5c). A Ramachandran Plot analysis verified that all the amino acid residues had correct and reasonable conformations in the model (Figure S5d).

### 4.3. Molecular Docking Simulation

All the SDF files for 3D chemical structure of ligands were obtained from PubChem Public Chemical Database and were transformed into PDB format with Chimera software. Then, PDB files of both ligand and laccase were used with Autodock software (Version 1.5.6, MGLTools, La Jolla, CA, USA) to generate PDBQT files for AutoGrid and AutoDock. Blind docking was done by setting Space value as 0.553 and x, y, z to be 126. Specific docking was done by centering at the T1 Copper (0.728, 5.862, 13.760) with Space value as 0.373 and x, y, z to be 70, 126, 70, respectively. After each docking run, a DLG file was obtained for analysis. Only the conformation with the lowest binding energy was visualized with Chimera. Interactions between ligand and amino acids were analyzed with the Ligplot v.1.4.5 software.

### 4.4. Reaction Tests

Reaction velocities (v_o_) were determined by mixing 50 μL of the properly diluted enzyme, 50 μL of substrate solution, and 2.4 mL of the media containing phosphate–citrate buffer (30 mM) and ILs in a 1 cm × 1 cm × 4 cm quartz colorimetric cell. An Agilent 8453A UV/Vis spectrophotometer with a magnetic stirrer accessory was used to record the initial velocities (v_o_) of ABTS at 420 nm (ε = 36,000 M^−1^·cm^−1^), 2,6-DMP at 468 nm (ε = 49,600 M^−1^·cm^−1^), syringaldazine at 525 nm (ε = 65,000 M^−1^·cm^−1^), and guaiacol at 470 nm (ε = 6740 M^−1^·cm^−1^) within 2 min. All the reported data were averaged from at least three parallel measurements.

### 4.5. Inhibition Kinetics

A series of v_o_ were measured under varied concentrations of substrate ([S]) and IL ([I]). The Lineweaver–Burk diagrams were then constructed by plotting 1/v_o_ against 1/[S] to estimate K_m_ and V_max_ according to Equation (1). The features of inhibition by different ILs were also deduced from the diagrams. K_i_ was correspondingly calculated from Equations (2), (3) or (4).

(1) $$(\mathrm{Lineweaver}–\mathrm{Burk})\;\frac{1}{v_{0}}=\frac{K_{M}}{V_{\max}\left[S\right]}+\;\frac{1}{V_{\max}}$$

(2) $$\left(\mathrm{Competitive}\right)\;\frac{1}{v_{0}}=\frac{K_{M}}{V_{\max}\left[S\right]}\left(1+\frac{\left[I\right]}{K_{ic}}\right)+\;\frac{1}{V_{\max}}$$

(3) $$\left(\mathrm{Noncompetitive}\right)\;\frac{1}{v_{0}}=\frac{K_{M}}{V_{\max}\left[S\right]}\left(1+\frac{\left[I\right]}{K_{in}}\right)+\;\frac{1}{V_{\max}}\left(1+\frac{\left[I\right]}{K_{in}}\right)$$

(4) $$\left(\mathrm{Uncompetitive}\right)\;\frac{1}{v_{0}}=\frac{K_{M}}{V_{\max}\left[S\right]}+\;\frac{1}{V_{\max}}\left(1+\frac{\left[I\right]}{K_{iu}}\right)$$

### 4.6. UV/Vis Spectroscopy of the Laccase/IL Complex

Laccase preparation with initial activity of 20 U was mixed with 50 mM [Bmim]Cl at 25 °C for 1 h. Then, the mixtures were directly freeze-dried to remove water. The obtained solids were washed with absolute ethanol 6 times. Centrifugation was performed to remove loosely bound ILs as well as other contaminants soluble in ethanol. The resultant solids were re-dissolved in 10 mL water. Then UV/Vis spectra were measured using water as blank. The reference sample was obtained following exactly the same process without addition of any IL.

### 4.7. Stability of ABTS Cation Radicals

ABTS cation radicals (ABTS^+^) were obtained from the oxidation of ABTS (10 mmol/L) with the laccase (50 U/L) in 50 μmol/L phosphate–citrate buffer (pH 3) at 40 °C for 2 h. Then, the reaction mixtures were centrifuged in an ultrafiltration tube (molecular weight cutoff 10 kDa; Millipore, Bedford, MA, USA) at 9000× *g* for 10 min). Enzymes and polymerized substances were removed. The oxidized products were incubated with buffer (30 mM, pH 2~7) and ionic liquids (50 mM) at 25 °C for 6 min. The concentration of each product was monitored by the spectrophotometer.

The first-order kinetics model was assumed to describe the time-dependent scavenging of ABTS^+^, which generally showed an exponential decrease in the plot with linear axes (Figure S6). The variation of ABTS concentration, Ln(A_o_)-Ln(A_t_), versus time (t) gave straight lines (Equation (5)). Half-life (*t*_1/2_) was a constant value and could be estimated from the rate constant (K) according to Equation (6).

(5) $$Ln\left(A\right)=Ln\left(A_{0}\right)-Kt/2.303$$

(6) $$t_{1/2}=K/2.303$$

## 5. Conclusions

This work provided new insights into the Hofmeister effects of alkylimidazolium ionic liquids (ILs) on fungal laccase activity. The alkylimidazolium cations competitively bound to the T_I_ Cu active pocket. Small cations with shorter alkyl chains (C_2_~C_6_) favourably entered the channel inside the pocket through hydrophobic interactions, exhibiting a good compatibility with laccase. Meanwhile, the species with long alkyl chains (C_8_~C_10_) formed stereo hindrance by occupying several key amino acids and finally led to a significant inhibition, i.e., at sites Leu296/Leu297 and Leu429. Complexation with alkylimidazolium cations caused a right shift of the laccase pH optima, thereby resulting in the invalidation of Hofmeister series of anions. In particular, EtSO_4_^−^ could bind near the T_I_ Cu active pocket, change the surrounding electric distribution and finally prevented the competitive binding of alkylimidazolium cations. In addition, alkylimidazolium ILs could accelerate the scavenging of ABTS^+^. However, it may not play a dominant role in the inhibitory effects of ILs.

## Figures and Tables

**Table 1:** Chemical structures of ionic liquids and substrates investigated in this work.

**Cations**					
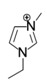	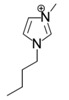	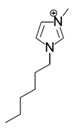	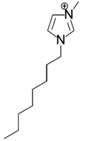	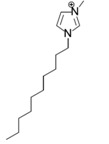	
[Emim]^+^	[Bmim]^+^	[Hmim]^+^	[Omim]^+^	[Dmim]^+^	
**Anions**		**Substrates**			
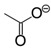	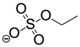	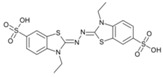	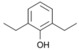	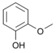	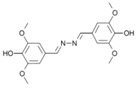
Ac^−^	EtSO_4_^−^	2,2’-azino-bis-(3-ethylbenzothiazoline-6-sulphonic acid) (ABTS)	2,6-dimethoxyphenol (2,6-DMP)	Guaiacol	Syringaldezine

**Table 2:** Optimal pH and relative activity of laccase towards different substrates.

Substrate	Buffer	[Emim]EtSO_4_	[Emim]Ac	[Bmim]Ac	[Emim]Cl	[Bmim]Cl
ABTS	pH 3 */ 100%	pH 3/ 95.9 ± 3.1%	pH 3/ 77.3 ± 0. 8%	pH 3/ 74.9 ± 1.3%	pH 3/ 72.1 ± 0.4%	pH 3/ 69.6 ± 0.2%
Syringaldazine	pH 5 *~6/ 100~94.1%	pH5~6/ 94.5~100.3%	pH5.5~6.5/ 96.9~98.3%	pH5.5~6.5/ 100.7~100.8%	pH 6/ 70.6 ± 2.7%	pH 6/ 79.7 ± 3.1%
2,6-DMP(I)	pH 2.5 */ 100%	pH 2.5~3/ 84.3~84.8%	pH 3/ 61.9 ± 2.7%	pH 2.5~3.5/ 67.4~77.3%	pH 2.5~3/ 4.5~4.7%	pH 2.5~3/ 4.3~4.5%
2,6-DMP(II)	pH 5 *~6/ 100~96.8%	pH 5~6/ 95.4~106.4%	pH 5.5~6.5/ 103.3~115.8%	pH 5.5~6.5/ 97.8~107.1%	pH 6/ 102.2 ± 0.3%	pH 6/ 102.9 ± 0.4%
Guaiacol	pH5 *~6/ 100~83.0%	pH5~6/ 99.4~93.7%	pH 5.5~6.5/ 89.55~80.01%	pH 5.5~6.5/ 96.4~82.4%	pH 6/ 38.9 ± 0.4%	pH 6/ 42.4 ± 0.9%

* Note: Normalized with the respective buffer control at optimal pH.

## Supplementary Materials

Supplementary materials are available online.

## Abbreviations

[Emim]^+^	1-ethyl-3-methyl-imidazolium
[Bmim]^+^	1-butyl-3-methyl-imidazolium
[Hmim]^+^	1-hexyl-3-methyl-imidazolium
[Omim]^+^	1-octyl-3-methyl-imidazolium
[Dmim]^+^	1-decyl-3-methyl-imidazolium
EtSO_4_^−^	ethylsulfate
AOT^−^	sulfosuccinate
Ac^−^	acetate
[TMA]TfO	tetramethylammonium trifluoromethanesulfonate
SDBS	sodium dodecyl benzene sulfonate
ABTS	2,2′-azino-bis-(3-ethylbenzothiazoline-6-sulphonic acid)
2,6-DMP	2,6-dimethoxyphenol
