# Supplementary material for: Molecular Mechanisms Underlying Inhibitory Binding of Alkylimidazolium Ionic Liquids to Laccase
Source: Molecules. 2017 Aug 15;22(8):1353. doi: 10.3390/molecules22081353 (PMC6152407; doi:10.3390/molecules22081353)
Supplement: Supplementary file 1 [file molecules-22-01353-s001.pdf]

Supplementary List

Figure S1. Lineweaver-Burk plots in presence of **o**ther four alkylimidazolium chlorides

(a) [Bmim]Cl, (b) [Hmim]Cl, (c) [Omim]Cl, (d) [Dmim]Cl

Figure S2. Binding of syringaldazine, 2,6-DMP and guaiacol with the T_I Cu active pocket of *Mth* laccase

(a) syringaldazine, (b) 2,6-DMP, (c) guaiacol

Figure S3. Scavenging of 2,6-DMP oxidative radicals in presence of [Bmim]Cl

Figure S4. Change of media pH by alkylimidazolium ILs

(Note: Buffer concentration 30 mM)

Figure S5. Homology modeling of *Myceliophthora thermophila* laccase

(a) Amino acids sequences, (b) 3D model, (c) QMEAN Z-score, (d) Ramachandran Plot

Figure S6. Plots of Ln[A_0]-Ln[A_1] vs. radical scavenging time

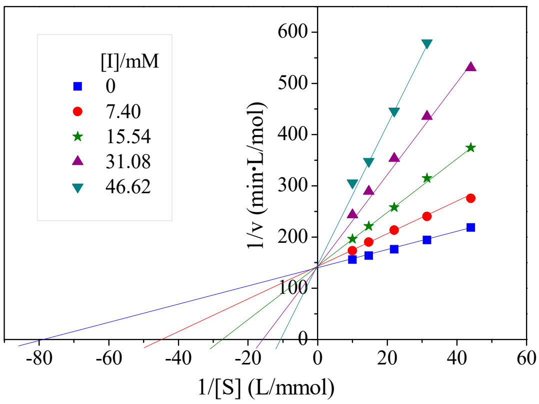

(a) [Bmim]Cl

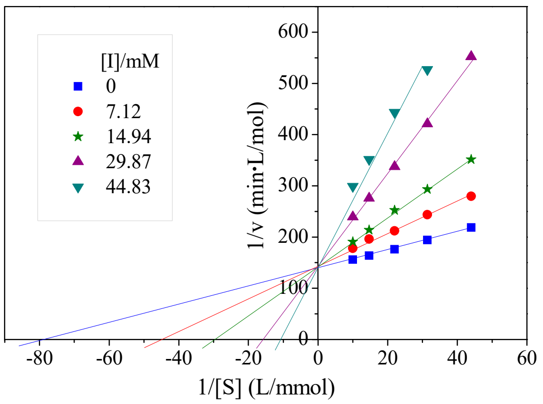

(b) [Hmim]Cl

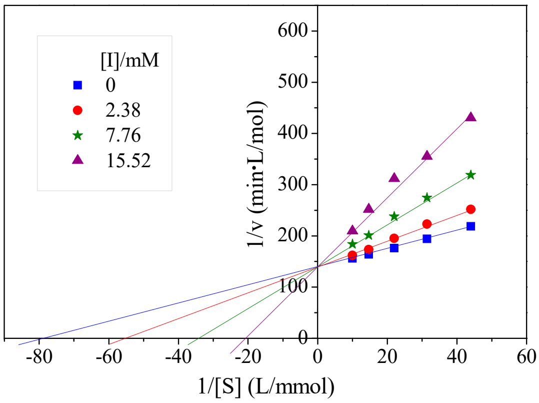

(c) [Omim]C

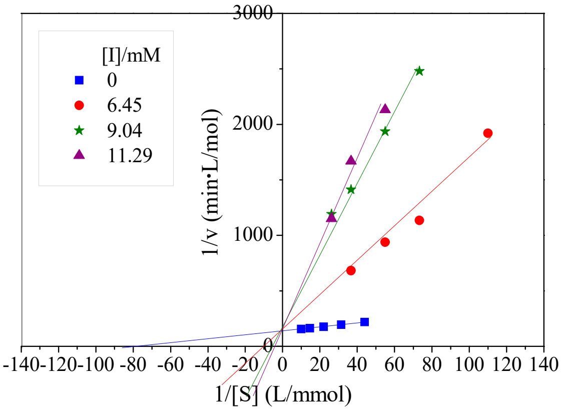

(d) [Dmim]Cl

Figure S1 Lineweaver-Burk plots in presence of other four alkylimidazolium chlorides。

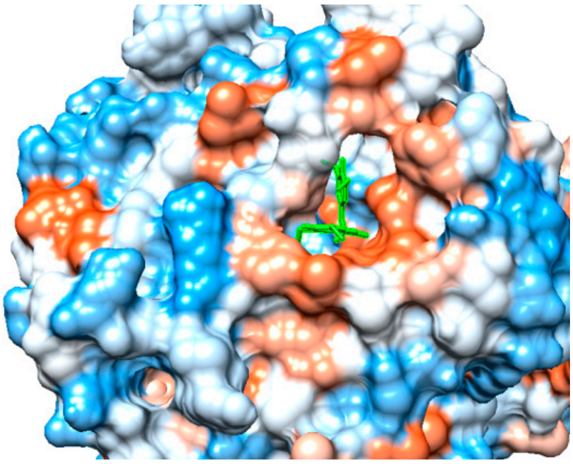

(a) Syringaldazine

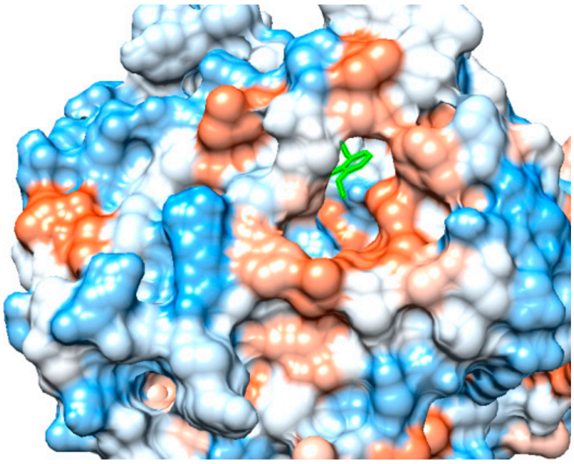

(b) 2,6-DMP

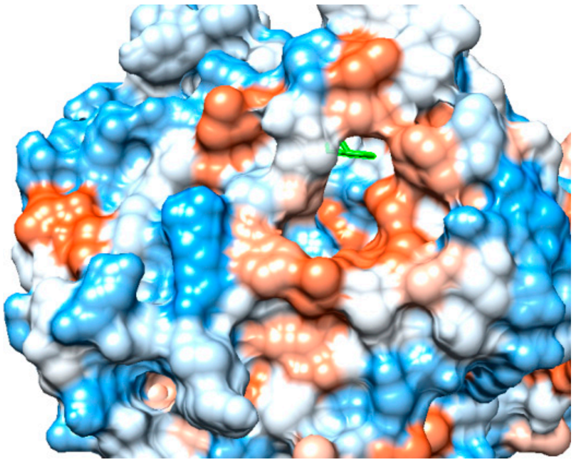

(c) Guaiacol

Figure S2. Binding of syringaldazine, 2,6-DMP and guaiacol with the T_1 Cu active pocket of *Mth* laccase

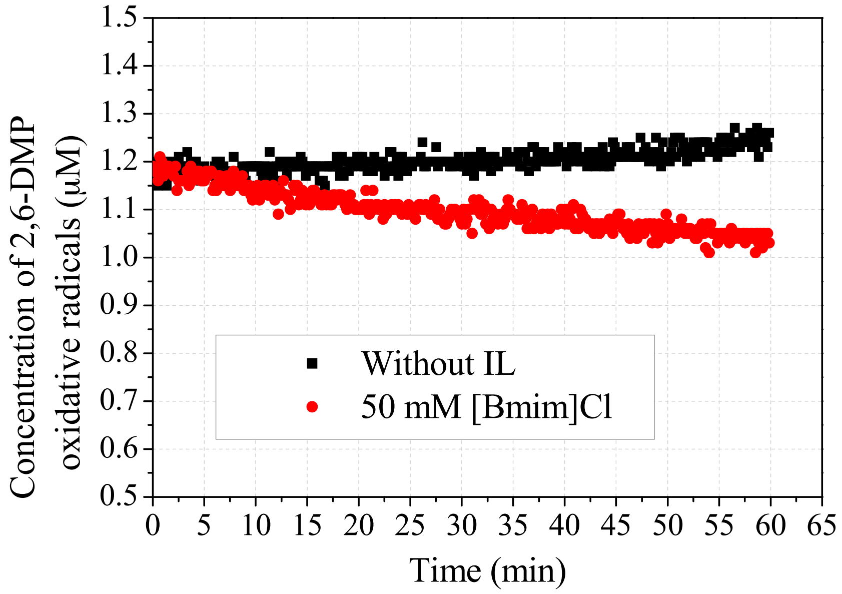

Figure S3. Scavenging of 2,6-DMP oxidative radicals in presence of [Bmim]Cl.

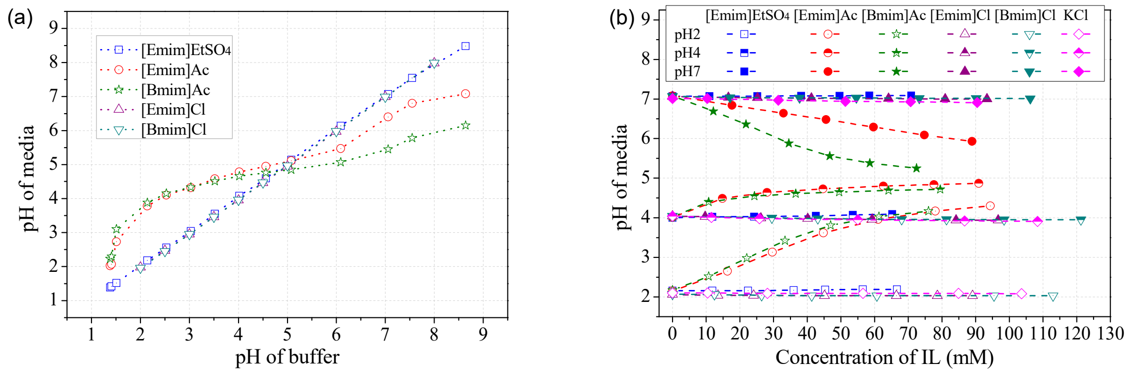

Figure S4. Change of media pH by different alkylimidazolium ILs (Note: Buffer concentration 30 mM).

Myceliophthora thermophila
(*Mth*) laccase

QQSCNTPSNRACWTDGYDINTDYEVDSPDTGVVRPYTLTLTEVDNWTGPDGVVKEKVMLVNNSIIGPTIF
ADWGDTIQVTVINNLETNGTSIHWHGLHQKGTNLHDGANGITECPIPPKGGRKVYRFKAQQYGTSWYHSH
FSAQYGNGVVGAIQINGPASLPYDTDLGVFPISDYYYSSADELVELTKNSGAPFSDNVLFNGTAKHPETG
EGEYANVTLTPGRRHRLRLINTSVENHFQVSLVNHTMCIIAADMVPVNAMTVDSLFLGVGQRYDVVIEAN
RTPGNYWFNVTFGGGLLCGGSRNPYPAAIFHYAGAPGGPPTDEGKAPVDHNCLDLPNLKPVVARDVPLSG
FAKRADNTLDVTLDTTGTPLFVWKVNGSAINIDWGRAVVDYVLTQNTSFPPGYNIVEVNGADQWSYWLIE
NDPGAPFTLPHPMHLHGHDFYVLGRSPDESPASNERHVFDPARDAGLLSGANPVRRDVSMLPAFGWVVLS
FRADNPGAWLFHCHIAWHVSGGLGVVYLERADDLRGAVSDADADDLDRLCADWRRYWPTNPYPKSDSGLK
HRWVEEGEWLVKA

Melanocarpus albomyces
(*Mal*) laccase

EPTCNTPSNRACWSDGFDINTDYEVSTPDTGVTQSYVFNLTEVDNWMGPDGVVKEKVMLINGNIMGPNIV
ANWGDTVEVTVINNLVTNGTSIHWHGIHQKDTNLHDGANGVTECPIPPKGGQRTYRWRARQYGTSWYHSH
FSAQYGNGVVGTIQINGPASLPYDIDLGVFPITDYYYRAADDLVHFTQNNAPPFSDNVLINGTAVNPNTG
EGQYANVTLTPGKRHRLRILNTSTENHFQVSLVNHTMTVIAADMVPVNAMTVDSLFLAVGQRYDVVIDAS
RAPDNYWFNVTFGGQAACGGSLNPHPAAIFHYAGAPGGLPTDEGTPPVDHQCLDTLDVRPVVPRSVPVNS
FVKRPDNTLPVALDLTGTPLFVWKVNGSDINVDWGKPIIDYILTGNTSYPVSDNIVQVDAVDQWTYWLIE
NDPEGPFSLPHPMHLHGHDFLVLGRSPDVPAASQQRFVFDPAVDLARLNGDNPPRRDTTMLPAGGWLLLA
FRTDNPGAWLFHCHIAWHVSGGLSVDFLERPADLRQRISQEDEDDFNRVCDEWRAYWPTNPYPKIDSGL

(a) Amino acids sequences

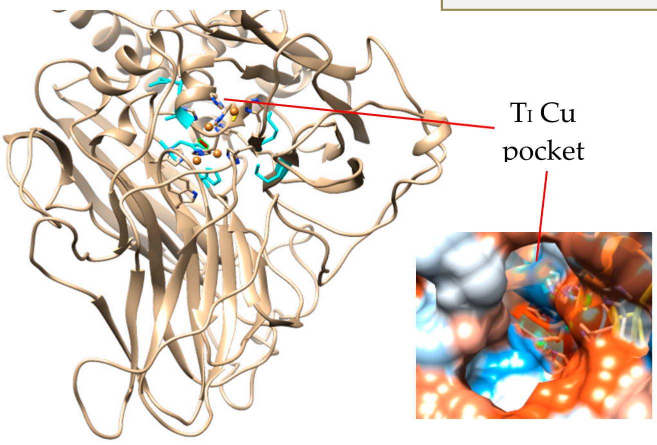

(b) 3D model

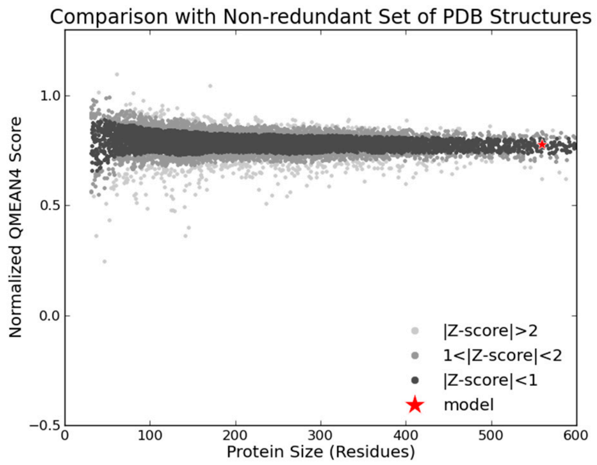

(c) QMEAN Z-score

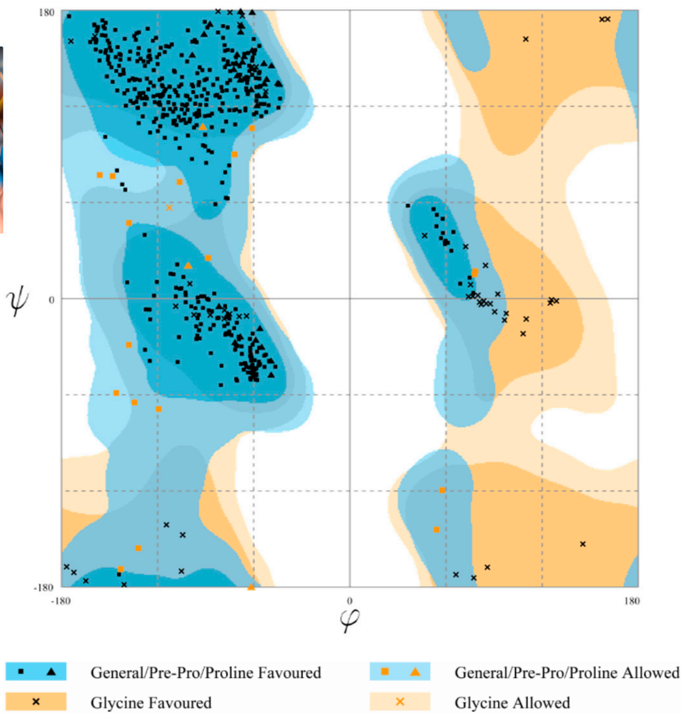

(d) Ramachandran Plot

Figure S5. Homology modeling of *Myceliophthora thermophila* laccase

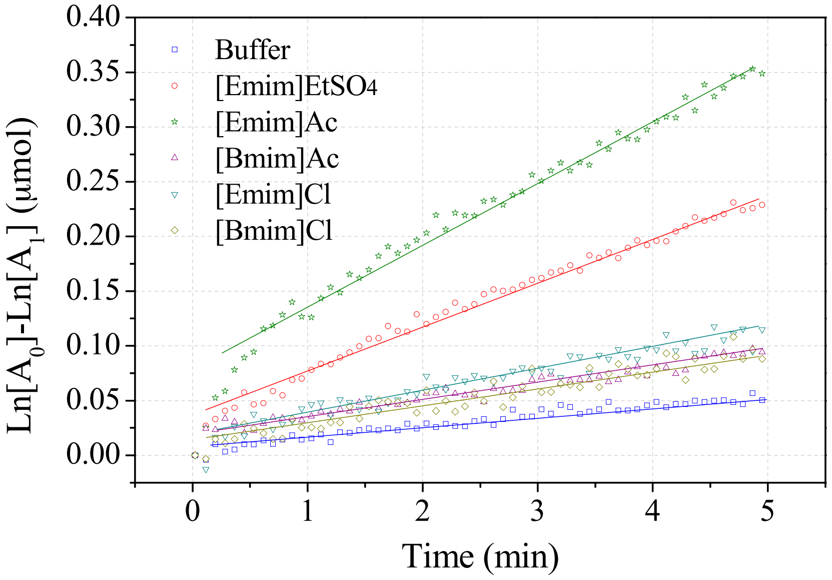

Figure S6. Plots of $Ln[A_0]$-$Ln[A_1]$ vs. radical scavenging time
